# Supplementary material for: Network Analysis of Genome-Wide Selective Constraint Reveals a Gene Network Active in Early Fetal Brain Intolerant of Mutation
Source: PLoS Genet. 2016 Jun 15;12(6):e1006121. doi: 10.1371/journal.pgen.1006121 (PMC4909280; doi:10.1371/journal.pgen.1006121)
Supplement: S1 Table — The mutational constraint signals (genes) in the top subnetwork show the significance clustering in terms of the number of nodes and edges and the clustering coefficient, and the constraint score sum against null expectation suggesting they function together. (PDF) [file pgen.1006121.s001.pdf]

Measures	P values	Observed
Node #	0.001	72
Edge #	0.001	146
Clustering coefficient	0.008	0.355
Constraint score	0.001	509.6
