# Supplementary material for: Network Analysis of Genome-Wide Selective Constraint Reveals a Gene Network Active in Early Fetal Brain Intolerant of Mutation
Source: PLoS Genet. 2016 Jun 15;12(6):e1006121. doi: 10.1371/journal.pgen.1006121 (PMC4909280; doi:10.1371/journal.pgen.1006121)
Supplement: S2 Table — Shown in the table are the p values of all significant tissues at a nominal significance (p = 0.05). Fetal brain shows consistently strong signal across all the threshold values. It suggests that fetal brain is most likely tissue of action. (PDF) [file pgen.1006121.s002.pdf]

Sig. Tissues	Fetal brain		CD3		CD8		CD34		Fetal thymus		Fetal heart	
Thres.	Null model 1	Null model 2	Null model 1	Null model 2	Null model 1	Null model 2	Null model 1	Null model 2	Null model 1	Null model 2	Null model 1	Null model 2
0.5	0	0	0	0.01	0.123	0.099	0.149	0.196	0.181	0.395	0.318	0.344
0.4	0	0	0.001	0.022	0	0.01	0.035	0.14	0.322	0.526	0.003	0.034
0.3	0	0	0	0.004	0	0.008	0	0.004	0.393	0.732	0.04	0.278
0.2	0	0	0	0.034	0	0.008	0	0	0.372	0.85	0	0.016
0.1	0	0.01	0.008	0.104	0	0.016	0	0	0	0.047	0.001	0.148
