# Supplementary material for: Network Analysis of Genome-Wide Selective Constraint Reveals a Gene Network Active in Early Fetal Brain Intolerant of Mutation
Source: PLoS Genet. 2016 Jun 15;12(6):e1006121. doi: 10.1371/journal.pgen.1006121 (PMC4909280; doi:10.1371/journal.pgen.1006121)
Supplement: S3 Table — Shown in the table includes: i) the number of tissue specific genes in the top subnetwork across the threseholds for all significant tissues. ii) the average number of tissue specific genes in the top subnetwork as well as entire network. iii) The median and standard deviation of tissue specific genes per tissues. iv) the mean and the standard deviation of tissue specificity of all genes. (PDF) [file pgen.1006121.s003.pdf]

	Number of tissue specific genes in the top subnetwork						Number of tissue specific genes in the entire network				Tissue specificity per gene	
Thres	Fetal brain	CD3	CD8	CD34	Fetal thymus	Fetal heart	Avg. across sig. Tissue	Avg. across all Tissue	Median across tissue	Standard dev. across tissue	Mean	Standard dev.
0.5	9	3	2	1	1	3	3.2	1.5	319	114.5	0.9 (3.1)	2.2 (3.1)
0.4	12	4	4	3	1	4	4.7	2.1	457	145	1.3 (3.5)	2.7 (3.5)
0.3	14	6	5	8	1	6	6.7	3	600	186.2	1.6 (3.9)	3.1 (3.8)
0.2	15	7	8	10	2	9	8.5	4.3	760	237.9	2.0 (4.2)	3.5 (4.1)
0.1	16	9	10	10	5	10	10	6.7	927	287.8	2.5 (4.6)	4.0 (4.4)
