# Supplementary material for: Network Analysis of Genome-Wide Selective Constraint Reveals a Gene Network Active in Early Fetal Brain Intolerant of Mutation
Source: PLoS Genet. 2016 Jun 15;12(6):e1006121. doi: 10.1371/journal.pgen.1006121 (PMC4909280; doi:10.1371/journal.pgen.1006121)
Supplement: S4 Table — We do not see significant overlap between the 72 genes in our constrained network that are preferentially expressed in fetal brain with those preferentially expressed in immune-system related cell types. This suggests that the tissue specific action of fetal brain is independent from that of immune-system related cell/tissue types. (PDF) [file pgen.1006121.s004.pdf]

Preferential expression threshold		CD8	CD34	Fetal thymus	Fetal heart	CD3
0.1	Fetal brain	0.39	0.39	0.07	na	na
	CD8		2.3E-04	9.5E-04	na	na
	CD34			0.14	na	na
	Fetal thymus				na	na
	Fetal heart					na
0.2	Fetal brain	0.21	0.34	na	1.8E-03	0.15
	CD8		8.3E-04	na	0.06	0
	CD34			na	0.02	3.3E-04
	Fetal thymus				na	na
	Fetal heart					0.04
0.3	Fetal brain	0.25	0.18	na	na	0.08
	CD8		4.0E-06	na	na	0
	CD34			na	na	2.3E-05
	Fetal thymus				na	na
	Fetal heart					na
0.4	Fetal brain	0.13	na	na	4.8E-04	0.13
	CD8		na	na	0.21	0
	CD34			na	na	na
	Fetal thymus				na	na
	Fetal heart					0.21
0.5	Fetal brain	na	na	na	na	0.04
	CD8		na	na	na	na
	CD34			na	na	na
	Fetal thymus				na	na
	Fetal heart					na
