# Supplementary material for: Network Analysis of Genome-Wide Selective Constraint Reveals a Gene Network Active in Early Fetal Brain Intolerant of Mutation
Source: PLoS Genet. 2016 Jun 15;12(6):e1006121. doi: 10.1371/journal.pgen.1006121 (PMC4909280; doi:10.1371/journal.pgen.1006121)
Supplement: S5 Table — All genes in top subnetwork, tissue-specific genes in the significant tissues such as fetal brain/CD34/CD8/fetal thymus, and all genome-wide significant mutational constraint genes. The total counts indicate the number of genes identified to have OMIM entries through biomarRt R package. The numbers in parenthesis indicate the actual total number of gene sets. (PDF) [file pgen.1006121.s005.pdf]

| Gene set | Total counts | In OMIM | Not in OMIM | P-values |
| --- | --- | --- | --- | --- |
| Roadmap epigenome | 9701 (9729) | 1989 | 7712 | NA |
| Top subnetwork | 71 (72) | 26 | 45 | 0.0013 |
| Fetal brain (TS) | 15 (16) | 6 | 9 | 0.068 |
| CD34 (TS) | 10 | 5 | 5 | 0.036 |
| CD8 (TS) | 10 | 4 | 6 | 0.13 |
| Fetal thymus (TS) | 5 | 2 | 3 | 0.273 |
| All significant genes | 124 (126) | 40 | 84 | 0.00155 |
