# Supplementary material for: Network Analysis of Genome-Wide Selective Constraint Reveals a Gene Network Active in Early Fetal Brain Intolerant of Mutation
Source: PLoS Genet. 2016 Jun 15;12(6):e1006121. doi: 10.1371/journal.pgen.1006121 (PMC4909280; doi:10.1371/journal.pgen.1006121)
Supplement: S6 Table — Among all the disease categories mapped to by at least one gene, only the nervous system disease shows significance. (PDF) [file pgen.1006121.s006.pdf]

| Gene set | Roadmap | Top subnetwork | P-values |
|---|---|---|---|
| Total # | 9729 | 72 | NA |
| Disease classified genes | 1455 | 19 | 0.0081 |
| Nervous System Diseases | 554 | 14 | 0.0017 |
| Eye Diseases | 162 | 2 | 0.64 |
| Male Urogenital Diseases | 122 | 1 | 0.81 |
| Female Urogenital Diseases and Pregnancy Complications | 131 | 2 | 0.52 |
| Cardiovascular Diseases | 149 | 3 | 0.3 |
| Hemic and Lymphatic Diseases | 153 | 1 | 0.88 |
| Congenital, Hereditary, and Neonatal Diseases and Abnormalities | 969 | 15 | 0.18 |
| Skin and Connective Tissue Diseases | 195 | 2 | 0.75 |
| Nutritional and Metabolic Diseases | 414 | 2 | 0.98 |
| Endocrine System Diseases | 144 | 2 | 0.57 |
| Pathological Conditions, Signs and Symptoms | 269 | 6 | 0.12 |
