# Supplementary material for: Network Analysis of Genome-Wide Selective Constraint Reveals a Gene Network Active in Early Fetal Brain Intolerant of Mutation
Source: PLoS Genet. 2016 Jun 15;12(6):e1006121. doi: 10.1371/journal.pgen.1006121 (PMC4909280; doi:10.1371/journal.pgen.1006121)
Supplement: S7 Table — Each column represents the following: Column 1: Curated pathway name; Column 2: Number of genes in each pathway; Column 3: Number of genes that are mapped to InWeb PPI network; Column 4: Number of genes in top subnetwork; Column 5: p-value of Gene Set Enrichment Analysis (Kolmogrov-Smirnov test); Column 6: p-value of hypergeometric test (PDF) [file pgen.1006121.s007.pdf]

Name	All	Mapped	Subnetwork	KS	OV
REACTOME_DEVELOPMENTAL_BIOLOGY	397	344	10	2.53E-19	1.83E-05
REACTOME_IMMUNE_SYSTEM	934	702	9	4.98E-08	0.019561975
REACTOME_ADAPTIVE_IMMUNE_SYSTEM	540	421	8	3.13E-10	0.002079468
REACTOME_AXON_GUIDANCE	252	220	8	4.62E-16	1.65E-05
REACTOME_MRNA_PROCESSING	162	120	8	9.06E-12	1.05E-07
KEGG_CALCIUM_SIGNALING_PATHWAY	179	163	7	6.00E-08	1.36E-05
KEGG_SPLICEOSOME	129	85	7	5.32E-21	9.60E-08
REACTOME_MRNA_SPLICING	112	74	7	6.26E-20	3.19E-08
REACTOME_PROCESSING_OF_CAPPED_INTRON_CONTAINING_PRE_MRNA	141	102	7	3.21E-13	3.99E-07
KEGG_PATHWAYS_IN_CANCER	329	301	6	4.05E-12	0.004205154
KEGG_REGULATION_OF_ACTIN_CYTOSKELETON	217	188	6	8.08E-13	0.00027377
REACTOME_CELL_CYCLE	422	332	6	0.007953708	0.007123284
REACTOME_MRNA_SPLICING_MINOR_PATHWAY	46	20	6	2.90E-05	3.56E-11
REACTOME_SIGNALLING_BY_NGF	218	191	6	3.04E-21	0.000301509
KEGG_FOCAL_ADHESION	202	188	5	9.72E-07	0.001695729
KEGG_LONG_TERM_POTENTIATION	71	60	5	9.64E-15	2.97E-06
KEGG_MAPK_SIGNALING_PATHWAY	268	233	5	3.02E-15	0.004917745
REACTOME_HIV_INFECTION	208	163	5	1.32E-06	0.00081242
REACTOME_HIV_LIFE_CYCLE	126	95	5	0.010256569	4.27E-05
REACTOME_LATE_PHASE_OF_HIV_LIFE_CYCLE	105	85	5	0.013577625	2.27E-05
REACTOME_NEURONAL_SYSTEM	280	219	5	3.80E-28	0.003636422
REACTOME_NGF_SIGNALLING_VIA_TRKA_FROM_THE_PLASMA_MEMBRANE	138	120	5	1.09E-14	0.000157186
REACTOME_SIGNALING_BY_GPCR	921	415	5	1.63E-11	0.06210229
