# Supplementary material for: Network Analysis of Genome-Wide Selective Constraint Reveals a Gene Network Active in Early Fetal Brain Intolerant of Mutation
Source: PLoS Genet. 2016 Jun 15;12(6):e1006121. doi: 10.1371/journal.pgen.1006121 (PMC4909280; doi:10.1371/journal.pgen.1006121)
Supplement: S8 Table — (PDF) [file pgen.1006121.s008.pdf]

Ensemble	HUGO	Sig.	Roadmap					BrainSpan - HIP			BrainSpan - VF				BrainSpan- PCW		
			fBrain	CD3	CD8	CD34	fHeart	HIP_2A	HIP_2B	HIP_3A	CGE_2A	LGE_2A	MGE_2A	STR_3A	PCx_2A	S1C_3A	IPC_3A
ENSG00000174231	PRPF8	1	0	0	0	0	0	1	0	1	1	1	1	1	1	1	1
ENSG00000197102	DYNC1H1	1	1	0	0	0	0	0	0	0	0	0	0	0	0	0	0
ENSG00000086758	HUWE1	1	0	0	0	0	0	0	0	0	0	0	0	0	0	0	0
ENSG00000127616	SMARCA4	1	1	0	0	0	0	1	1	1	1	1	1	1	1	1	1
ENSG00000181222	POLR2A	1	0	0	0	0	0	0	0	0	0	0	0	0	0	0	0
ENSG00000198626	RYR2	1	1	0	0	0	1	0	0	0	0	0	0	0	0	0	0
ENSG00000184634	MED12	1	0	0	0	1	0	1	1	1	1	1	1	1	1	1	1
ENSG00000144028	SNRNP200	1	0	0	0	0	0	1	1	1	1	1	1	1	1	1	1
ENSG00000111642	CHD4	1	0	0	0	0	0	1	1	1	1	1	1	0	1	0	0
ENSG00000198793	MTOR	1	0	0	0	0	0	0	0	0	0	0	0	0	0	0	0
ENSG00000176884	GRIN1	1	0	0	0	0	0	0	0	0	0	0	0	0	0	0	0
ENSG00000177380	PPFIA3	1	0	0	0	0	0	0	0	0	0	0	0	0	0	0	0
ENSG00000118058	MLL	1	1	0	0	0	0	0	0	0	0	0	0	0	0	0	0
ENSG00000104517	UBR5	1	0	0	0	0	0	0	0	0	0	0	0	0	0	0	0
ENSG00000150995	ITPR1	1	0	0	0	1	0	0	0	0	0	0	0	0	0	0	0
ENSG00000141367	CLTC	1	0	0	0	0	0	0	0	0	0	0	0	0	0	0	0
ENSG00000196924	FLNA	1	0	0	1	0	0	0	0	0	0	0	0	0	0	0	0
ENSG00000005007	UPF1	1	0	0	0	0	0	0	0	0	0	0	0	0	0	0	0
ENSG00000172534	HCFC1	1	0	0	0	0	0	0	0	0	0	0	0	0	0	0	0
ENSG00000132153	DHX30	1	0	0	0	0	0	0	0	0	0	0	0	0	0	0	0
ENSG00000115306	SPTBN1	1	1	1	0	0	1	0	0	0	0	0	0	0	0	0	0
ENSG00000115524	SF3B1	1	0	0	0	0	0	0	0	0	0	0	0	0	0	0	0
ENSG00000080503	SMARCA2	1	0	1	1	1	0	0	0	0	0	0	0	0	0	1	1
ENSG00000100346	CACNA1I	1	0	0	0	0	0	0	0	0	0	0	0	0	0	0	0
ENSG00000072501	SMC1A	1	0	0	0	0	0	1	1	1	1	1	1	1	1	1	1
ENSG00000150086	GRIN2B	1	1	0	0	0	0	0	1	1	0	1	1	1	0	1	1
ENSG00000105464	GRIN2D	1	0	0	0	0	0	0	0	0	0	0	0	0	0	0	0
ENSG00000147133	TAF1	1	0	0	0	0	0	0	0	0	0	0	0	0	0	0	0
ENSG00000165280	VCP	1	0	0	0	0	0	0	0	0	0	0	0	0	0	0	0
ENSG00000125107	CNOT1	1	0	0	0	0	0	1	1	1	1	1	1	1	1	1	1
ENSG00000038382	TRIO	1	1	0	0	0	1	0	0	0	0	0	0	1	0	1	1
ENSG00000055163	CYFIP2	1	1	1	1	1	1	0	0	0	0	0	0	0	0	0	0
ENSG00000196235	SUPT5H	1	0	0	0	0	0	0	0	0	0	0	0	0	0	0	0
ENSG00000177283	FZD8	1	0	0	0	0	0	0	1	1	1	1	1	0	1	0	0
ENSG00000105576	TNPO2	1	1	0	0	0	0	0	0	0	0	0	0	0	0	0	0
ENSG00000077809	GTF2I	1	1	0	0	0	0	0	0	0	0	0	0	0	0	0	0
ENSG00000127481	UBR4	1	0	0	0	0	1	0	0	0	0	0	0	0	0	0	0
ENSG00000170004	CHD3	1	1	1	1	0	0	0	0	0	0	0	0	1	1	1	1
ENSG00000187555	USP7	1	0	1	1	0	0	0	0	0	0	0	0	0	0	0	0
ENSG00000101161	PRPF6	1	0	0	0	1	0	0	0	0	0	0	0	1	0	1	1
ENSG00000087460	GNAS	1	0	0	0	0	0	0	0	0	0	0	0	0	0	0	0
ENSG00000125676	THOC2	1	0	0	0	0	0	0	1	1	1	1	1	1	1	0	0
ENSG00000073910	FRY	1	1	0	0	1	1	0	0	0	0	0	0	1	0	1	1
ENSG00000147162	OGT	1	0	0	0	0	0	0	0	0	0	0	0	0	0	0	0
ENSG00000047315	POLR2B	1	0	0	0	0	0	1	1	1	1	1	0	1	1	1	1
ENSG00000156113	KCNMA1	1	1	0	0	0	0	0	0	0	0	0	0	0	0	0	0
ENSG00000160551	TAOK1	1	0	0	0	0	0	0	0	0	0	0	0	0	0	0	0
ENSG00000165288	BRWD3	1	0	0	0	0	0	1	1	1	1	1	1	1	1	1	1
ENSG00000197694	SPTAN1	1	1	0	0	0	1	0	0	0	0	0	0	0	0	1	1
ENSG00000146247	PHIP	1	0	0	0	0	0	1	1	1	1	1	1	0	1	0	0
ENSG00000167986	DDB1	1	0	0	0	0	0	1	0	0	1	0	0	0	0	0	0
ENSG00000126803	HSPA2	1	0	0	0	0	0	0	0	0	0	0	0	0	0	0	0
ENSG00000072195	SPEG	1	0	0	0	0	0	0	0	0	0	0	0	0	0	0	0
ENSG00000108055	SMC3	1	0	0	0	0	0	1	1	1	1	1	1	1	1	1	1
ENSG00000133026	MYH10	1	1	0	0	0	1	1	0	0	1	1	1	0	1	0	0
ENSG00000082898	XPO1	1	0	0	0	0	0	0	1	1	1	1	1	1	0	0	0
ENSG00000036257	CUL3	1	0	0	0	0	0	0	0	0	1	1	0	0	0	0	0
ENSG00000185950	IRS2	1	0	0	0	0	0	0	0	0	0	0	0	0	0	0	0
ENSG00000164742	ADCY1	1	1	0	0	0	0	0	0	0	0	0	0	0	0	1	1
ENSG00000115266	APC2	1	0	0	0	0	0	0	0	0	0	0	0	0	0	0	0
ENSG00000116809	ZBTB17	1	0	0	0	0	0	0	0	0	0	0	0	0	0	1	0
ENSG00000137076	TLN1	1	0	0	1	1	0	0	0	0	0	0	0	0	0	0	0
ENSG00000100345	MYH9	1	0	1	1	0	0	0	0	0	0	0	0	0	0	0	0
ENSG00000167658	EEF2	1	0	0	0	1	0	1	1	1	1	1	1	1	1	1	1
ENSG00000121892	PDS5A	1	0	0	0	0	0	1	1	0	1	0	1	0	0	0	0
ENSG00000105287	PRKD2	1	0	1	1	0	0	1	0	1	1	1	1	0	1	0	0
ENSG00000141867	BRD4	1	0	0	0	0	0	0	0	0	0	0	0	0	0	0	0
ENSG00000109971	HSPA8	0	0	0	0	0	0	0	0	0	0	0	0	0	0	0	0
ENSG00000168036	CTNNB1	0	0	0	0	0	1	1	1	1	0	0	0	0	1	0	0
ENSG00000150991	UBC	0	0	0	0	0	0	0	0	0	0	0	0	0	0	0	0
ENSG00000171608	PIK3CD	0	0	1	1	1	0	0	0	0	0	0	0	0	0	1	1
ENSG00000145675	PIK3R1	0	0	1	1	1	1	0	1	1	0	0	0	0	0	0	0
