# Supplementary figures and images for: Network Analysis of Genome-Wide Selective Constraint Reveals a Gene Network Active in Early Fetal Brain Intolerant of Mutation
Source: PLoS Genet. 2016 Jun 15;12(6):e1006121. doi: 10.1371/journal.pgen.1006121 (PMC4909280; doi:10.1371/journal.pgen.1006121)

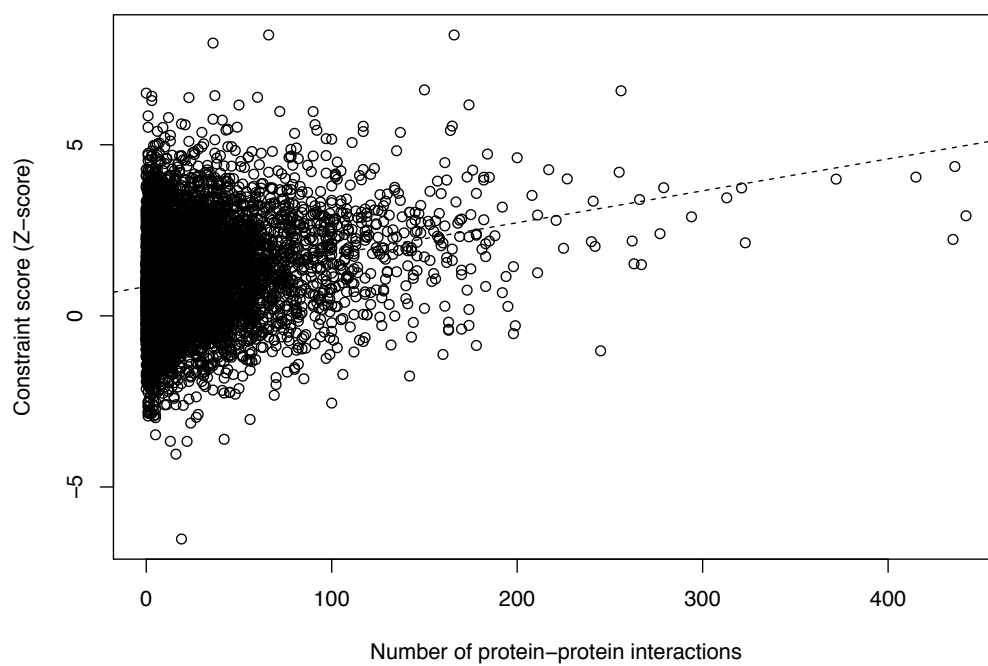

Supplement: S1 Fig — Constraint Z scores are only weakly correlated with InWEB node degree. Pearson correlation coefficient = 0.022. (PDF) [file pgen.1006121.s009.pdf]
