# Supplementary material for: Network Analysis of Genome-Wide Selective Constraint Reveals a Gene Network Active in Early Fetal Brain Intolerant of Mutation
Source: PLoS Genet. 2016 Jun 15;12(6):e1006121. doi: 10.1371/journal.pgen.1006121 (PMC4909280; doi:10.1371/journal.pgen.1006121)
Supplement: S1 Text — (PDF) [file pgen.1006121.s010.pdf]

Supplementary information

Supplementary note

Supplementary methods

1. Permutation method for subnetwork detection significance testing
2. Threshold dependence analysis of significant tissues
3. Tissue specific gene overlap among significant tissues
4. Online Man In Mendelian (OMIM) entry enrichment analysis
5. Medical Subject Headings (MeSH) disease category enrichment analysis
6. Pathway enrichment analysis

Supplementary note

During the completion of this project, the constraint score analysis was updated from ~61,000 individuals in Samocha *et al* 2014 to over 91,000 included in the Exome Aggregation Consortium (exac.broadinstitute.org, Lek *et al*, to appear; http://biorxiv.org/content/early/2015/10/30/030338). Though a full re-analysis of these data is beyond the scope of this manuscript, our main observation of a constrained network of 72 genes remains unchanged.

Supplementary methods

1. Permutation method for subnetwork detection significance testing

We tested the significance of the clustering of mutational constraint signals in the detected top subnetwork against an empirical null expectation. To generate the null expectation, we repeated the subnetwork search algorithm (Step 2 of PINTS workflow) 1000 times with random permutations of mutational constraint scores across the InWeb PPI network, which yielded 1000 null sets of top subnetwork (null top subnetworks). The significance of the clustering is evaluated comparing the number of nodes, the number of edges, clustering coefficient, and the constraint score sum of the top subnetwork with those of 1000 null top subnetworks (see Supplementary table 1).

2. Threshold dependence analysis of significant tissues

We repeated the tissue specificity analysis of the top subnetwork over the different tissue specificity threshold values. The threshold values considered ranges from 10 to 50 percentile of maximum preferential expression value (PE+) in 10 percentile steps. In this range, the average number of tissue specific genes within the top subnetwork varies 1.5 (at the 50 percentile) to 6.7 (at the10 percentile) across all tissues (see Supplementary table 2 and 3).

3. Tissue specific gene overlap among significant tissues

To examine if different tissue/cell types show a significant overlap or form distinct subcomponents in the top subnetworks, we tested the significance of overlap between tissue specific genes among all significant tissues over the different thresholds using a hypergeometric test (Supplementary table 4).

4. Online Man In Mendelian (OMIM) entry enrichment analysis

To test OMIM [1] entry enrichment of genes in the top subnetwork, we used Fisher’s exact test comparing the proportion of genes that have OMIM entries with those that do not have OMIM entries. We used biomaRt R package to retrieve OMIM records for all the genes identified to have transcript-level expression value after the pre-processing of Roadmap epigenomics exon array dataset (Supplementary table 5).

5. Medical Subject Headings (MeSH) disease category enrichment analysis
To test MeSH disease category enrichment, we first mapped OMIM entries to MeSH disease categories using Comparative Toxicogenomics Database’s (CTD) MEDIC disease vocabulary [2]. We then used Fisher's exact test comparing the proportion of genes that are classified to a particular disease category with those that are not classified to the same category (Supplementary table 6).

6. Pathway enrichment analysis
To test pathway enrichment, we used the GSEA approach [3]. The curated canonical pathways (c2.cp.v4.0.symbols.gmt) are downloaded from GSEA website: http://www.broadinstitute.org/gsea/msigdb/collections.jsp. We also tested the significance of overlap between all genes in the pathways mapped to InWeb PPI network and those in the top subnetwork using a hypergeometric test (Supplementary table 7).
